# Autonomous vehicles with augmented reality internet of things and edge intelligence system for industry 5.0 based on 6G

**DOI:** 10.1371/journal.pone.0339022

**Published:** 2025-12-18

**Authors:** Amjed A. Ahmed, Aqeel Kamil Kadhim, Mohammad Kamrul Hasan, Sumaia M. AL-Ghuribi, Dhafar Hamed Abd, Salah A. Aliesawi, Belal Abdullah Hezam Murshed, Luke Topham, Wasiq Khan, Abir Jaafar Hussain

**Affiliations:** 1 University of Imam Al-Kadhum, Baghdad, Iraq; 2 Department of Computer Techniques Engineering, Imam Alkadhum College (IKC), Baghdad, Iraq; 3 Center for Cyber Security, Faculty of Information Science and Technology at Universiti Kebangsaan Malaysia (UKM), Bangi, Malaysia; 4 Software Engineering Department, College of Computer Engineering & Sciences, Prince Sattam Bin Abdulaziz University, Alkharj, Saudi Arabia; 5 College of Computer Science and Information Technology, University of Anbar Ramadi, Ramadi, Iraq; 6 Faculty of Engineering and Information Technology, Amran University, Amran City, Yemen; 7 School of Computer Science and Mathematics, Liverpool John Moores University, Liverpool, United Kingdom; 8 College of Engineering, University of Sharjah, Sharjah, United Arab Emirates; Alma Mater Studiorum Universita di Bologna: Universita degli Studi di Bologna, ITALY

## Abstract

In an era of rapidly evolving technology, traditional cloud computing struggles to meet the demands of resource-intensive smart devices. This necessitates a shift towards Edge Computing (EC), which brings computation and data storage closer to the network’s edge, enhancing efficiency and reducing latency. This is particularly crucial for the Internet of Things (IoT), where supporting mobility, location awareness, and real-time processing are paramount. However, the scalability of EC applications is significantly influenced by network parameters and the capabilities of the computing system. This paper proposes a novel system architecture for Industry 5.0 that leverages the synergy between 6G networks, autonomous vehicles, Augmented Reality (AR), IoT, and edge intelligence to revolutionize transportation systems. Our approach integrates AR for enhanced user interfaces, utilizes IoT for data acquisition and control, and employs edge computing for real-time decision-making. Our experimental results demonstrate a strong correlation between processing speed and network bandwidth. While increasing either parameter individually enhances overall system performance. The two-tier architecture, combined with the Entity Objects (EO) model, demonstrates superior scalability compared to traditional approaches. By distributing processing tasks and leveraging the resources of other edge servers, the system can handle increasing numbers of AVs and data loads without compromising performance.

## 1. Introduction

The integration of automation, computer systems, controls, and communication in Intelligent Transportation Systems (ITS) [1] aims to enhance transportation sustainability, efficiency, and safety. Autonomous vehicles (AVs) [2] have garnered significant attention as an ITS that prioritizes surface-level mobility. AVs rely on a plethora of technologies, including electronics, vehicle dynamics, communication, control systems, sensing, and traffic pattern analysis. With AVs poised to replace human drivers, manual car control becomes obsolete. Decades of continuous research, fuelled by substantial investments from global technology companies, have paved the way for the age of autonomous vehicles. Leveraging the Internet of Things (IoT) [3,4], edge intelligence [5], blockchain smart contracts [6], and vehicular networks [7], AVs can meet consumer demands for features like self-verification, self-execution, immutability, data reliability, and security. AVs offer a promising solution to transportation’s economic and environmental challenges by mitigating traffic congestion, accidents, and pollution.

Notably, human error is a significant contributor to 93% of accidents, often resulting from factors such as drunk driving or speeding [8]. AVs, with their reduced reliance on human drivers, have the potential to reduce accident rates drastically. Research by the Insurance Institute for Highway Safety [9] indicates that safety features like lane departure warnings, side view assist, and frontal collision avoidance can significantly decrease vehicle-related injuries and fatalities by 33%. Furthermore, AVs can empower individuals who are unable to drive due to age, disability, or other limitations, thereby enhancing their quality of life and social inclusion. Simultaneously, AVs can optimize fuel consumption, fleet management, commuting efficiency, and overall traffic conditions [10].

Despite the potential benefits, developing Level 5 autonomous vehicles faces technical hurdles and ongoing investigations into trust and safety concerns. On-road testing by companies like Google, Tesla, Audi, BMW, Mercedes-Benz, and others contributes to research on AV architectural bottlenecks [11]. However, a more modern statistical approach is needed to analyze and address these challenges [12]. Further research is crucial to enhance the safety and reliability of AVs, accelerating their commercial viability.

The sixth generation of wireless technology (6G) promises unprecedented speeds, ultra-low latency, and extensive interconnectivity. Integrating 6G with autonomous vehicles, Augmented Reality (AR), the IoT, and edge intelligence has the potential to revolutionize the Industry 5.0 landscape. Autonomous vehicles stand to benefit significantly from advancements in 6G. Real-time data transfer is vital for their navigation, collision avoidance, and decision-making capabilities. 6G networks will enable seamless communication between AVs and surrounding infrastructure, leading to safer and more efficient transportation systems.

AR is poised to become a crucial element in enhancing the user experience within autonomous vehicles. By seamlessly blending digital information with the real world, AR provides drivers and passengers with critical information and situational awareness. 6G’s high responsiveness and ultra-fast speeds enable AR applications to deliver interactive content such as notifications, guidance, and alerts, ultimately contributing to a safer and more intuitive driving experience.

The proliferation of smart devices further expands the capabilities of Automated Transport and Real-time Visualization Systems. As mobile technology increasingly integrates into transportation ecosystems, networked devices can optimize data collection, track environmental parameters, and facilitate communication. 6G technology enhances these capabilities by enabling efficient processing of large datasets, providing high responsiveness to commands, and improving communication performance. This empowers devices to exhibit enhanced perception and decision-making for vehicles and infrastructure.

Edge intelligence plays a crucial role in optimizing networked devices, particularly in enhancing the responsiveness of automated systems and minimizing data transmission latency. By processing data locally at the network edge, coupled with 6G’s ultra-fast connectivity, data exchange between edge devices, central servers, and other nodes becomes seamless. This facilitates real-time adaptability and decision-making for highly autonomous vehicles and AR applications.

In the proposed framework, autonomous vehicles, augmented reality (AR), the Internet of Things (IoT), and edge intelligence all work together within a 6G network-supported environment, interacting in a manner that ensures fluid and intelligent navigation in an Industry 5.0 context. Autonomous vehicles are mobile nodes equipped with local sensors and processors that collect and transmit environmental and operational data locally. This information is disseminated through 6G to edge intelligence nodes, which perform rapid, localized analysis to enable capabilities such as instantaneous navigation, obstacle avoidance, and joint route planning. IoT-connected devices actively track the health of machines, their surroundings, and the location of assets, generating vast amounts of data at the edge layer to perform necessary analysis and provide a response as quickly as possible. AR interfaces combine both edge and 6G capabilities, overlaying relevant information (such as vehicle diagnostics or operational tips) onto a real-life view, enhancing the interaction between a human and a machine in real-time. The synergy of these components will lead to a highly responsive, adaptive, and intelligent system in which the decisions are made locally at the edge when required, and more complex operations are offloaded to the cloud, resulting in optimized performance, minimal latency, and robust behaviour of the system under the dynamic industrial environment.

### 1.1. Motivation

The need for this research stems from the emerging requirement for real-time, intelligent, and resilient systems in Industry 5.0, where human-centered collaboration with machines and cyber-physical systems is mandatory. Conventional architectures relying on cloud-based systems are unable to support the rigorous latency, bandwidth, and reliability requirements of current industrial settings, particularly as the number of autonomous vehicles, AR interfaces, and dense IoT networks is deployed. The advent of 6G technology, with its promises of providing low-latency, high-speed connectivity and the capability to support device densities an order of magnitude greater than current densities, affords a rare chance to overcome these constraints. The proposed architecture will enable an immersive yet dynamic industrial ecosystem by incorporating 6G, edge intelligence, and autonomous systems, along with immersive AR experiences. The convergence not only optimizes the effectiveness of real-time decision-making and operational efficiency but also overcomes the shortcomings of current models, ensuring scalability, security, and context awareness. This, in turn, has triggered the development of an integrated, next-generation framework specifically designed to handle Industry 5.0.

AR-based maintenance and training solutions further enhance industrial worker productivity and safety. AR headsets provide technicians with real-time instructions, schematics, and troubleshooting guides, enabling them to complete tasks faster and reduce downtime. These 6G-enabled AR applications deliver seamless user experiences regardless of location or environment, fostering a more agile and connected workforce.

The key contributions of our research are as follows:

- We propose a system that integrates IoT sensors, Augmented Reality, and edge intelligence to optimize resource utilization and energy efficiency.
- We conduct a comprehensive assessment of the impact of network and computing system parameters on the scalability and performance of edge environments, focusing on AR-enabled autonomous vehicles powered by 6G networks.
- Our research highlights how edge computing, facilitated by 6G, enables real-time data analysis and predictive maintenance, optimizing production schedules and minimizing the risk of costly breakdowns. This convergence of technologies paves the way for highly efficient and environmentally friendly smart factories.
- We demonstrate how the IoT network enables equipment monitoring, anomaly detection, and automated parameter adjustments to maximize uptime and reduce waste.
- Finally, we evaluate the performance of our proposed system in terms of task failure rate, network latency, and processing time.

The remainder of this paper is organized as follows. Section 2 explains the literature review. In section 3, we present our proposed system architecture and key parameters. Section 4 presents the experimental design work. The obtained results are discussed in Section 5. Finally, Section 6 presents the conclusion and future direction of our research work.

## 2. Related work

As an extension of the IoT, the Internet of Vehicles (IoV) [13] plays a crucial role in smart transportation applications. Numerous IoV frameworks offer infrastructure-level methods and tools for processing data from fog clouds and road services. Previous research has focused on secure data processing by cooperative vehicles in fog cloud networks. For instance, the authors of [14] aimed to develop a globally accessible control management system for the IoV to prevent collisions between vehicles in smart cities, specifically examining the utilization of road-unit services for vehicular applications. Their approach leverages centralized and decentralized cloud nodes to enable traffic prediction and location services within a smart city context. Mohammed MS et al. [15] proposed an intelligent Driving Assist System (DAS) for real-time prediction of steering angle using deep learning (DL) and a raw dataset collected from a real environment.

Robust malware detection systems are crucial for ensuring security at various nodes involved in vehicle data processing [16]. This study explored the potential of fog cloud networks for autonomous traffic forecasting and collision detection, emphasizing the importance of secure data offloading. Addressing vehicle collaboration, the authors of [17] proposed an Intelligent Intrusion Detection System (IIDS) integrated with Software Defined Networking (SDN) for cooperative vehicles. Similarly, [18] focused on securing data transmission between moving vehicles using peer-to-peer networks. However, while this approach enhances security, it introduces increased overhead due to the utilization of distributed network channels for managing secure data transfer between vehicles on a one-to-one basis. Moreover, most existing research on intrusion detection relies on static analysis, making IoV applications vulnerable to runtime attacks employing unknown patterns.

Leading automotive manufacturers like Tesla and Google have introduced several self-driving vehicles, highlighting the IoT’s transformative potential in realizing autonomous driving. The evolution of the IoT into the Internet of Autonomous Vehicles (IoAV) signifies a paradigm shift, transforming vehicles from mere objects into collaborative entities within a connected ecosystem [19]. This transition will eventually lead to autonomous agents assuming control of vehicles.

The exponential growth of smart devices and their increasing interconnectedness has led to a massive surge in data flow. While cloud computing offers significant storage capacity, it struggles to meet the low latency, rapid response time, and high Quality of Service requirements of real-time applications. Although advancements in fog, mobile cloud, and edge computing have mitigated some challenges, infrastructure and security concerns persist.

Artificial intelligence (AI) has emerged as a key enabler for achieving fully autonomous vehicles. Integrating AI with edge computing creates Edge Intelligence (EI), leveraging AI’s ability to analyze vast datasets and extract meaningful insights. However, the diversity of concepts and technologies involved in this integration presents significant challenges.

Over 50 billion interconnected smart devices are anticipated within the IoT ecosystem by 2030 [20]. This necessitates efficient data offloading to edge servers, seamless cooperation among them, and localized data analysis to alleviate the burden on distant cloud infrastructure. Advancements in vehicle-to-everything (V2X) communication, coupled with sophisticated AI and machine learning (ML) algorithms, have the potential to elevate vehicle perception, decision-making, and cognition to human-like levels, paving the way for full autonomy [21]. The emergence of 5G and 6G communication technologies will further enhance V2X capabilities, providing vehicles with a comprehensive understanding of their surroundings. These technologies aim to enable seamless and ultra-reliable low-latency communication between vehicles.

Given the complexity of autonomous vehicle infrastructure, security and privacy breaches pose significant risks to commuters and passengers [21]. With its decentralized nature, data immutability, and transparency, blockchain technology offers a promising solution for ensuring the security of autonomous vehicles. By effectively integrating and enhancing these technologies within a robust system, we can achieve the goal of fully autonomous driving.

The research focusing on the design aspect of integrating AVs with augmented reality, IoT, and Edge Intelligence has been discussed in previous works. However, there are still a few limitations. First, a significant portion of prior research [16–18] focuses on investigating the potential of individual technologies – for instance, AR for driving assistance or IoT for vehicle communication – while the integration of these elements, considering various facets of real-world contexts, is not sufficiently explored. Furthermore, most work [17–19] has focused on ubiquitous yet limited and relatively static networks, while the ability of these systems to operate in dynamic, large-scale transportation networks remains largely uninvestigated. The next challenge is the issue of computing power at the edge, where some dedicated devices for AR, real-time IoT data, and AI edge intelligence are not sufficiently powerful to support these tasks. Finally, most proposed systems overlook the energy efficiency of multi-layered technology, thereby underscoring the challenges involved in implementing these solutions in rational, self-sustaining commercial vehicles. Table 1 presents a comparison of the proposed work with existing literature in terms of classification.

**Table 1:** Classification of the proposed work in comparison with existing literature.

Author(s)	Technology Focus	Scope/Contribution	Limitation/Gap	Gap Bridged in this Work
Yang et al. (2021) [22]	Edge–Cloud for AVs	Task scheduling and offloading in AVs	No AR integration, limited to cloud–edge split	Introduces real-time AR interaction and edge intelligence fusion
Biswas et al. (2023) [1]	MEC with 5G for IoT vehicles	Improved latency in vehicular networks using mobile edge computing	Limited scalability, lacks 6G and AR-based feedback loops	Incorporates 6G, AR, and IoT synergy for adaptive vehicle systems
Kumar et al. (2022) [15]	Federated edge intelligence	Distributed learning at the edge in smart transport systems	Ignores human–machine interface and AR overlays	Adds AR-enabled user interface for real-time interaction
Mohammed et al. (2023) [22]	AR for navigation	Visual AR-based guidance for indoor/outdoor systems	Standalone AR, no vehicular integration or edge intelligence	Integrates AR into live AV control with edge offloading
Jameel et al. (2024) [20]	IoT in Industry 5.0	Connected devices for smart manufacturing and transportation	No autonomy, lacks vehicular context, and multi-layered orchestration	Combines AVs, IoT, and 6G with orchestration for Industry 5.0
Our work	AV + AR + IoT + Edge + 6G	Unified architecture for smart mobility in Industry 5.0	—	Full-stack, real-time, human-interactive autonomous vehicle model

**Fig 1:**
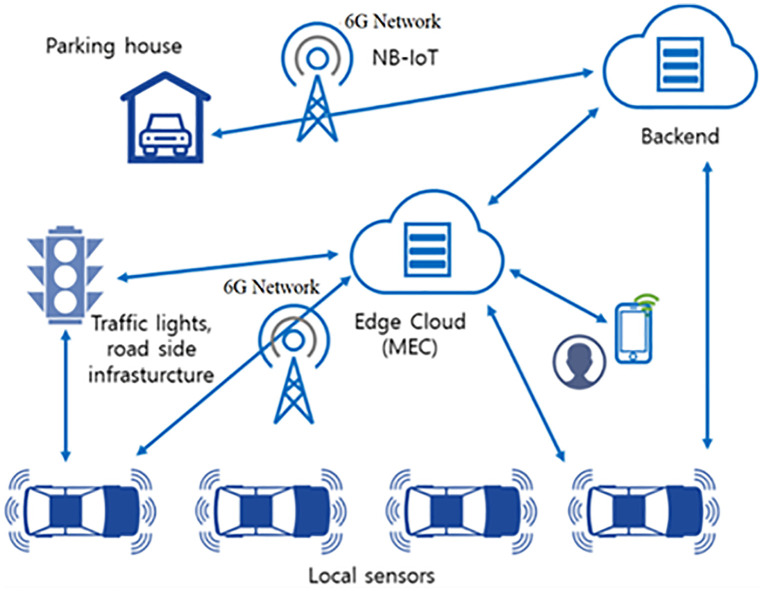
Integrated system architecture with key parameters of 6G and edge computing for autonomous vehicles.

This study aims to demonstrate how 6G technology can empower edge computing-based autonomous vehicles, as illustrated in Fig 1.

## 3. Methodology

This section presents the proposed system’s methodology, requirements for use cases, system design, and use case scenarios.

### 3.1. Method

**Fig 2:**
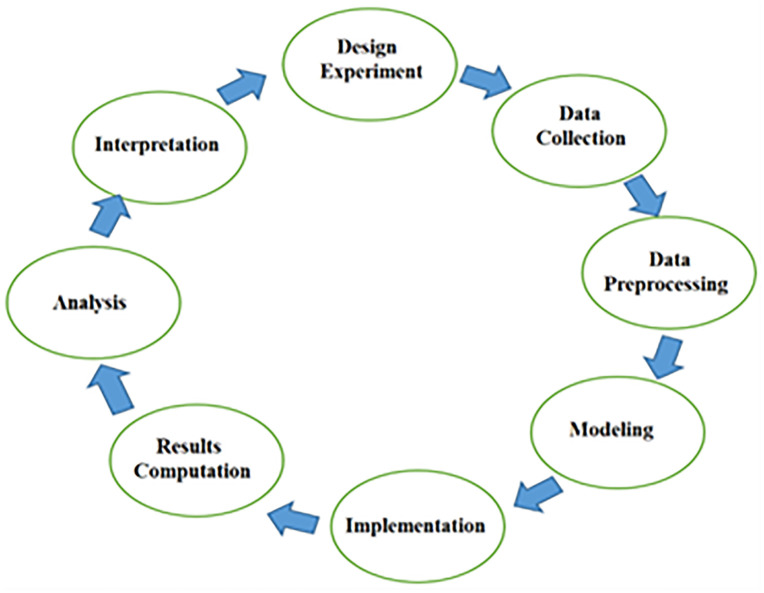
An overview of the Agile methodology.

This study employs an agile methodology, as illustrated in Fig 2. This approach allows for multiple iterations throughout the experimental phase. The agile methodology comprises several steps: (1) Design Experiment, (2) Data Collection, (3) Data Pre-processing, (4) Modeling, (5) Implementation, (6) Analysis, and (7) Interpretation. This iterative process facilitates both the initial design of the experiment and the subsequent analysis and interpretation of the results.

### 3.2. System architecture

This subsection describes the system architecture and key parameters. The Edge Computing (EC) environment consists of three levels. An Edge Layer comprises edge processors, Entity Objects (EOs), and the IoT devices layer, encompassing all IoT devices necessary to execute tasks. Edge Servers can access the cloud layer, enabling task offloading from the cloud data center. A Cloud Layer provides centralized resources and services.

Designing experiments requires considering various factors within this architecture. However, the EdgeCloudSim emulator imposes limitations on system architecture and layer configurations. This research focuses on three primary architectures for the edge layer. The first is a single-tier (Monolithic) centralized architecture, where all components reside on a single server. The second is a two-tier (Client-Server) approach, where end devices, such as IoT devices or mobile users, offload processing tasks to dedicated edge servers or the cloud. The third is two-tier with EO, similar to the two-tier architecture, but incorporates EOs to facilitate task offloading to additional edge servers or the cloud.

**Fig 3:**
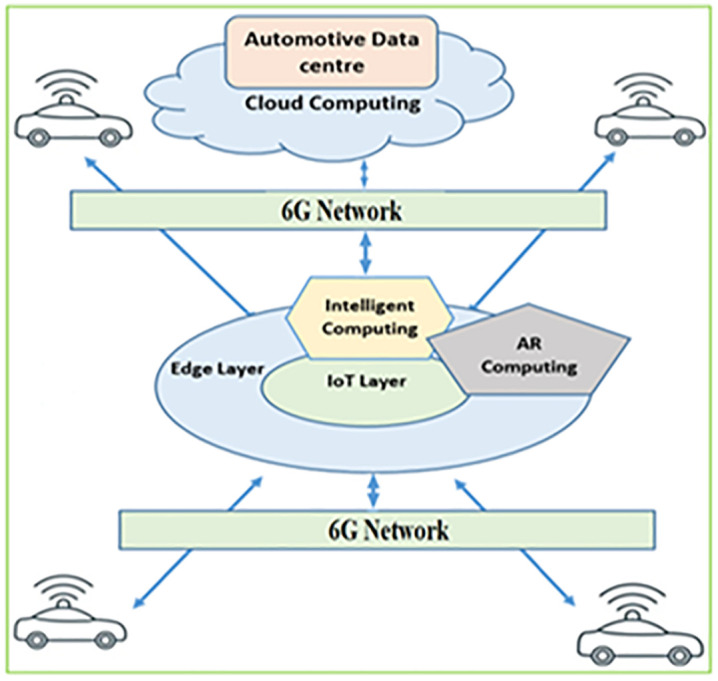
The architecture of Edge layers and 6G Network in the proposed model.

Fig 3 illustrates these proposed edge layer architectures. It highlights the essential components and actors to consider during experimentation. IoT Devices represent autonomous vehicles in our scenario. The application simulates an augmented reality app’s object recognition feature, requesting processing from autonomous vehicles. EO and Edge Administrators: EOs enable task offloading to edge servers while edge administrators manage resources and services. Cloud provides additional computing resources and services when required. Wi-Fi Access Point connects autonomous vehicles to the edge server or, in some cases, the WAN. EdgeCloudSim allows us to configure and experiment with these actors, components, and configurations.

The system architecture considers three aspects: Tier 1: Onboard Processing and Edge Intelligence, Tier 2: Networked Collaboration and Augmented Reality, and Entity Object (EO) Model.

Tier 1: Onboard Processing and Edge Intelligence. Unlike many conceptual designs, this architecture centers around AVs equipped with LiDAR, radar, and cameras for comprehensive environmental perception. These sensors collect real-time data on road conditions, obstacles, and traffic flow patterns. Crucially, the first tier emphasizes onboard processing through edge computing. Each AV possesses significant computational capacity, enabling real-time decision-making for navigation, control, and interaction with the surrounding environment. This edge intelligence minimizes reliance on centralized servers, reducing latency, a critical factor for safe and efficient AV operation.

Tier 2: Networked Collaboration and Augmented Reality. The second tier extends the system’s scope to a networked environment using 6G and IoT technologies. Roadside sensors, traffic signals, and other vehicles communicate with AVs in real-time, facilitating cooperative driving by sharing intentions, speed, and manoeuvres. This interconnectedness enhances overall traffic flow and safety. Furthermore, AR interfaces improve human-machine interaction within AVs. Situational awareness is significantly enhanced by superimposing information, such as navigation instructions, hazard warnings, and vehicle diagnostics, onto the driver’s field of view.

Entity Object (EO) Model. The proposed architecture utilizes an EO model to manage the system’s complexity. Each AV, along with other elements like traffic infrastructure and roadside units, is represented as an EO with its own data and processing capabilities. This model facilitates efficient data exchange and coordination between various system components, enabling seamless interaction between AVs and the surrounding smart infrastructure.

Table 2 provides a categorized summary of the architecture, where every core component is ordered according to its functionality, deployment, and interaction within the system. Such organized representation illustrates how the most cutting-edge trends, including 6G, edge computing, AR, IoT, and autonomous vehicles, can be used to achieve Industry 5.0 goals. The perception and device layer, which comprises IoT sensors and an AV system, serves as the foundation for real-time data collection and mobility. Furthermore, the processing and orchestration levels utilize edge computing, enabling smarter task assignment to achieve ultra-low-latency decision-making. The AR interface layer mediates human and machine intelligence, whereas the cloud layer controls the long-term storage and large-scale analysis. Such a stratified strategy depicts an arrangement that is highly integrated and effectively manages the dynamic environments of complex transportation. It describes the top-down structure of data and control flows, with inputs arriving at sensor-level, real-time edge processing, and potentially further synchronizing with the cloud. Top-down and bottom-up interaction pathways facilitate cross-layer communication, which will be enabled by the high-speed and high-reliability features of 6G networks. Since the relationships between system parts can be visualized in a layered model, it remains straightforward to determine which components rely on each other, including the interface between AR and edge-level processing of sensor data provided by IoT sensors, as well as the way orchestration at the edge distributes the load among connected AVs. Such multilevel classification not only helps define the role of each module but also illustrates the scalability, modularity, and adaptability of the architecture, and once again contributes to its aptitude for real-life implementation in smart transportation systems within the scope of Industry 5.0.

**Table 2:** Classification of Key Components in the Proposed Architecture.

Technology	Layer	Function	Deployment Level	Category
6G Communication	Network Layer	Ultra-low latency, high bandwidth connectivity	Infrastructure	Enabler
Autonomous Vehicle	Device Layer	Real-time mobility, decision-making, navigation	Edge Device	End System
Augmented Reality	Interface Layer	Visual feedback, user interaction, spatial guidance	User Interface	Human–Machine Layer
IoT Sensors	Perception Layer	Data acquisition (location, speed, environment)	Vehicle/Environment	Sensing Component
Edge Computing	Processing Layer	Local analytics, task offloading, and latency reduction	Roadside/Vehicle	Processing Platform
Cloud Computing	Storage Layer	Global data storage, historical analytics, and backups	Central Infrastructure	Backup/Storage
Edge Orchestration	Control Layer	Task allocation, load balancing, decision routing	Edge Gateway	Coordination Module

### 3.3. Scenarios

The following are three real-world use case scenarios. Scenario 1: Object Recognition in Autonomous Vehicles. Autonomous vehicles require robust object recognition capabilities to ensure safe navigation. In a two-tier (client-server) architecture, video streams from autonomous vehicles are transmitted to an edge server for analysis. The edge server identifies objects such as pedestrians, animals, and obstacles. If necessary, the video can be forwarded to another edge node or the cloud for further processing. This scenario highlights the importance of low-latency communication and efficient data processing at the edge.

Scenario 2: Mobility and Resource Management. EdgeCloudSim often employs the nomadic mobility model for autonomous vehicles. This model assumes that vehicles move within a defined area, spending varying amounts of time at different locations. For instance, vehicles typically spend more time in traffic circles than on straight roads. This factor is crucial in real-world scenarios, as task completion and offloading decisions depend on vehicle location and dwell time. Additionally, the availability and processing capacity of edge servers in each area must be considered to ensure the timely execution of tasks.

Scenario 3: Industry 5.0 Applications. 6G-enabled autonomous vehicles, integrated with AR, IoT, and edge intelligence, offer transformative potential for Industry 5.0 applications. These technologies can enhance safety, efficiency, and productivity in industrial settings.

Consider a scenario in which a fleet of autonomous vehicles, equipped with advanced sensors and onboard processing units, operates within a smart factory. These vehicles navigate autonomously, communicate in real-time with each other and control systems, and leverage 6G connectivity for seamless data exchange. Workers equipped with AR-enabled smart glasses can access real-time situational data and interact with machines more effectively. Sensors deployed throughout the factory collect data on environmental conditions, device status, and manufacturing processes. This data is transmitted via 6G to edge servers for real-time analysis and decision-making. Edge processing ensures low latency and enables prompt responses to events, optimizing system performance.

By leveraging these cutting-edge technologies, businesses can achieve unprecedented levels of productivity, agility, and competitiveness. Integrating advanced communication, sensor networks, and edge computing, this system lays the foundation for smart manufacturing and the realization of Industry 5.0.

The proposed architecture model exhibits flexibility in different network conditions by incorporating edge intelligence, network slicing in the 6G architecture, and dynamic system and orchestration to enforce real-time behavior and maintain scalability. Local processing of time-sensitive data is made possible by edge computing, which removes or minimizes dependence on centralized infrastructure during periods of latency or loss of connectivity. The 6G network slicing will enable the prioritization of bandwidth, ensuring that critical operations, such as autonomous navigation and AR-based assistance, are not disrupted. Also, offloading of computation between vehicles, edge nodes, and the cloud is supported in real-time based on network and processing demands. It also uses fallback communication modes (e.g., NB-IoT) and predictive network probing to enhance data flows (as well as service quality) on demand. All these adaptive mechanisms guarantee solid latency-insensitive performance, with system performance and responsiveness preserved despite variable network traffic.

The proposed system architecture addresses many of the main weaknesses of traditional cloud computing, including, but not limited to, high latency, bandwidth restrictions, and the lack of contextual awareness, by leveraging 6G-enabled edge intelligence and decentralized computing. Most traditional cloud models have poor real-time capabilities, as communication between data sources (e.g., autonomous vehicles, IoT devices) and centralized servers is a major source of delays and inefficiencies in high-stakes industrial processes. Conversely, the proposed architecture incorporates edge computing units capable of processing data locally, enabling ultra-low latency response times and direct decision-making, which are crucial to Industry 5.0 use cases such as autonomous navigation and AR interaction. The system platform is more flexible and adaptive than established edge computing (EC) models because it is synergistic with the features of 6G technologies, including network slicing, terahertz communication, and intelligent routing, which guarantee dynamic resource allocation and robust output. This architecture is more responsive than the cloud and poses less dependency on it, with low network congestion, and offers a distributed computing platform that creates a robust framework for computing highly compatible with high-speed and real-time industrial needs.

## 4. Experimental design

The experimental design follows an iterative approach, beginning with a well-defined experiment, followed by data collection, data pre-processing, analysis, and interpretation. The initial simulation duration is set to 30 minutes. However, a 60-minute simulation time is employed to capture critical architectural layer interactions due to hardware limitations that hinder continuous loading. To effectively evaluate system performance under various loading conditions, the number of autonomous vehicles gradually increases in 25% increments, starting from 25 and reaching a maximum of 300.

### 4.1. Experimental assumptions

The experiments operate under the following assumptions. (1) Asymmetric Data Traffic: We assume a significantly larger volume of uploaded data than downloaded data. Therefore, the upload data size is set to 2000 KB, while the download size is 15 KB. (2) Low Latency Requirement: Real-time object identification in autonomous vehicles necessitates low-latency communication. Consequently, the system is designed to prioritize low latency, with a delay sensitivity of 95%. (3) Short Stay Duration: Considering the fast-moving nature of autonomous vehicles, a stay duration of 60 seconds is assumed for most locations. However, recognizing that certain areas, such as traffic circles, may require longer dwell times, the second location in the simulation has a stay duration of 120 seconds.

These assumptions enhance the realism of the simulation environment. Table 3 summarizes the simulation parameters and their corresponding values.

**Table 3:** Values of the simulation parameters setup.

Parameters	Values
Simulation time	50 Min.
Uploading data	1 MB
Downloading data	20 KB
Interarrival task time	3 min.
Processing speed of the cloud	20100 MIPS
Active time	30 sec
Idle time	1 sec
Min. no. of autonomous vehicles	20
Max. no. of autonomous vehicles	150
No. of edge servers in location type1	4
No. of edge servers in location type2	8
WAN Bandwidth	35 Mbps
Wireless LAN Bandwidth	350 Mbps
Edge VM’s processing speed	2000 MIPS
No. of VMs per edge server	4

### 4.2. Experimental design

This subsection details the experimental design. The primary objective is to investigate the impact of various parameters on system performance. The parameters under investigation are Edge Server Density, WLAN Bandwidth Impact, Virtual Machine Processing Speed, and the Combined Impact of WLAN Bandwidth and VM Processing Speed.

The AR interface is built on top of the Unity 3D platform, powered by the AR Foundation system and Vuforia SDK, which allows it to run on mobile devices and head-mounted displays (like Microsoft HoloLens 2 and Magic Leap). The devices offer high-end SLAM features, as well as a real-time object tracking system, which is vital for visualizing navigation routes, issuing danger warnings, and conducting real-time system analysis. The AR module is directly linked to the edge intelligence layer, as it accepts sensor fusion input and outputs a dynamic overlay based on the output of obstacle detection and route planning. The first usability tests were conducted using the User Experience Questionnaire (UEQ), yielding favourable results in terms of clarity, efficiency, and responsiveness. Gesture recognition, the use of eye-tracking feedback, and pilot testing of the system in real-world environments will be introduced in future versions to maximize the effectiveness of human-machine interaction and ensure that the AR interface enhances, rather than detracts from, autonomous vehicle operation in Industry 5.0 surroundings.

Edge Server Density: This experiment examines the effect of deploying dedicated edge servers in each area. Eight edge servers are used to evaluate the impact of the bottleneck identified in the initial experiment. Increasing the number of edge servers is expected to reduce task failure rates. WLAN Bandwidth Impact: This experiment analyzes the influence of WLAN bandwidth on system performance, specifically its effect on the previously identified bottleneck. The bandwidth is gradually increased from 100 Mbps to 500 Mbps in increments of 100 Mbps. Virtual Machine Processing Speed: This experiment investigates the impact of virtual machine (VM) processing speed on the identified bottlenecks. Three sub-experiments are conducted, each with a different VM processing speed. Starting from 1000 MIPS, the speed is incremented by 1000 MIPS for each sub-experiment. Increasing the VM processing speed is anticipated to reduce task failure percentages. Combined Impact of WLAN Bandwidth and VM Processing Speed: This experiment evaluates the synergistic effect of increasing WLAN bandwidth and VM processing speed on the identified bottlenecks. Each sub-experiment involves a simultaneous increase of 100 Mbps in WLAN throughput and 1000 MIPS in VM processing speed. This combined approach is expected to significantly reduce task failures.

By systematically varying these parameters and analyzing their individual and combined effects, the experiments aim to identify optimal configurations that minimize task failures and enhance the overall performance of the proposed system. The corresponding algorithm is shown in Algorithm 1.

**Algorithm 1.** Proposed Integrated AV System with AR, IoT, and Edge Intelligence.

IntegratedAVSystem(AV, AR, IoT, EdgeNodes, Destination)

Step 1: Initialization

 Initialize(AV)

 Configure(PositioningSystem)

 Configure(AR_Display)

 Configure(IoT_Modules)

 Configure(EdgeNodes)

 Configure(Sensors)

Step 2: Set Destination

 Destination ← InputCoordinates()

 CurrentLocation ← GetAVLocation()

 Activate(NavigationSystem)

Step 3: Navigation Process

 while CurrentLocation ≠ Destination do

 NextMove ← ComputeNextStep(CurrentLocation, Destination)

 Obstacles ← DetectObstacles(Sensors)

 RoadStatus ← AnalyzeRoadConditions(Sensors)

 SendToIoT(SensorsData)

 CurrentLocation ← UpdateLocation()

Step 4: Sensor Data Processing

 SensorData ← Read(Sensors)

 ProcessedData ← Analyze(SensorData)

 Features ← ExtractFeatures(ProcessedData)

### 4.3. Implementation

The experiments are conducted using the EdgeCloudSim simulator. The simulation environment is set up on an Apple MacBook Pro laptop running macOS High Sierra build 10.13.5. The laptop is equipped with an Intel i5 processor and 8GB of RAM.

### 4.4. Evaluation metrics

Several performance metrics are used to evaluate the system. These metrics offer insights into the computational and network aspects that influence the scalability of the design. Utilizing multiple metrics allows for a comprehensive understanding of the relationship between specific outcomes and system parameters. The evaluation metrics employed are as follows. (1) Task Failure Rate: This metric measures the percentage of tasks that fail to complete successfully within a given timeframe. A lower task failure rate indicates better system performance. (2) Network Latency: This metric quantifies the delay experienced by data packets traveling across the network. Lower network latency is desirable for real-time applications, such as object identification in autonomous vehicles. (3) Processing Time: This metric measures the time taken by the system to process a given task. Shorter processing times indicate more efficient resource utilization and faster response times.

## 5. Experimental results and discussion

This section presents the experimental results, analyzes them in the context of two-tier and two-tier with Entity Objects (EO) architectures, and discusses the limitations of the proposed work.

### 5.1. Results analysis

This section presents the results of the proposed system. The data presented below outlines the computation of task processing, failure rates, and delays in a 2-tier computational system with and without Edge Optimization (EO), across varying workloads, and in the presence or absence of mobile devices. The experiments indicate that the computation at the edges will always outperform the classical two-tier system in terms of the extent to which tasks fail and the processing delivered. The population consists of 300 devices. Using the analysis presented in Tables 4 and 5, it is evident that the probability of failed tasks at the edge server and the cloud is significantly impacted when the number of devices increases. The 2-tier architecture incorporating EO achieves a relatively lower failure rate as a result of increased device density.

**Table 4:** Failed Tasks on Edge Server (%) [Edge Computing Environment].

Number of Mobile Devices	2-tier (%)	2-tier with EO (%)
25	10	5
50	25	15
75	40	25
100	55	35
125	70	50
150	80	60
175	85	65
200	90	70
225	95	75
250	98	80
275	99	85
300	100	90

**Table 5:** Failed Tasks on Cloud (%) [Cloud Computing Environment].

Number of Mobile Devices	2-tier (%)	2-tier with EO (%)
25	0	0
50	5	2
75	10	5
100	20	10
125	35	15
150	50	25
175	65	35
200	75	45
225	85	55
250	90	65
275	95	75
300	100	85

In practice, where the guiding principle is the efficient movement of the vehicle, autonomy has quite dynamic movement dynamics, which can be disrupted by unpredictable traffic, traffic stop times, and differences in city infrastructure. In addition, unexpected external signals also include impacts on sensor performance and AR rendering by environmental noise, sensor interference, and unfavourable weather conditions that may considerably affect sensor accuracy, AR rendering, and communication reliability. Additional risks are associated with system failures, which can be as significant as edge node disconnection through IoT devices or signal corruption in 6G networks. To address these complexities, future improvements will include stochastic modelling of vehicle mobility, adaptive dwell time estimation, and resilient task offloading strategies. Additionally, strong error-handling systems and on-the-fly fault detection systems should be incorporated to ensure the framework aligns more closely with real Industry 5.0, thereby maintaining the stability of performance and overall system continuity despite operational uncertainties.

The results presented in Tables 6 and 7 show that EO significantly assists in reducing overall processing time for both edge and cloud networks. Specifically, the proposed 2-tier system with EO details a significant decrease on the edge server side while preserving process timeframe fluctuation in line with the rising number of devices.

**Table 6:** Average Processing Time on Edge Server (Seconds) [Edge Computing Environment].

Number of Mobile Devices	2-tier (s)	2-tier with EO (s)
25	0.4	0.2
50	0.8	0.5
75	1.1	0.8
100	1.3	1.0
125	1.5	1.1
150	1.6	1.2
175	1.6	1.2
200	1.6	1.2
225	1.7	1.3
250	1.7	1.3
275	1.7	1.3
300	1.7	1.3

**Table 7:** Average Processing Time on Cloud (Seconds) [Cloud Computing Environment].

Number of Mobile Devices	2-tier (s)	2-tier with EO (s)
25-300	0.15	0.12

The findings reported in Tables 8 and 9 show that the mean network delay, in terms of WLAN and WAN, decreases when the 2-tier system includes Edge Optimization (EO). As shown above, EO has less delay than the standard 2-tier, indicating that more efficient networks will occur for all device counts and no bottleneck will occur in either edge or cloud computation environments.

**Table 8:** Average WLAN Delay (Seconds) [Edge Computing Environment].

Number of Mobile Devices	2-tier (s)	2-tier with EO (s)
25	0.05	0.04
50	0.08	0.05
75	0.12	0.07
100	0.15	0.10
125	0.18	0.12
150	0.15	0.10
175	0.12	0.08
200	0.10	0.07
225	0.14	0.08
250	0.18	0.10
275	0.20	0.12
300	0.18	0.11

**Table 9:** Average WAN Delay (Seonds) [Cloud Computing Environment].

Number of Mobile Devices	2-tier (s)	2-tier with EO (s)
25	0.2	0.1
50	0.3	0.2
75	0.5	0.3
100	0.7	0.5
125	0.9	0.7
150	1.0	0.8
175	1.1	0.9
200	1.2	1.0
225	1.3	1.1
250	1.4	1.2
275	1.5	1.3
300	1.6	1.4

As illustrated in Tables 10 and 11, in the succeeding subsections, the research reveals the moderating influence of EO on the rate of failed tasks and the slowdown of combination in a network. Although the failure rate remains constant on the edge server for many mobile devices, the 2-tier system with EO significantly reduces failure rates through intelligent computational distribution. The WLAN and WAN delays are measured to give an added dimension to the efficiency of EO.

**Table 10:** Failed Tasks on Edge Server (%) [Edge Computing Environment].

Number of Mobile Devices	2-tier (%)	2-tier with EO (%)
25	5	2
50	15	10
75	30	20
100	45	35
125	60	50
150	75	65
175	85	75
200	90	80
225	95	85
250	98	90
275	99	92
300	100	95

**Table 11:** Failed Tasks on Cloud (%) [Cloud Computing Environment].

Number of Mobile Devices	2-tier (%)	2-tier with EO (%)
25	0	0
50	0	0
75	0	0
100	1	0
125	5	2
150	10	5
175	20	10
200	35	15
225	50	25
250	70	35
275	85	50
300	95	60

As demonstrated by the results in Tables 12 and 13, the WLAN latencies are still significantly lower with EO, which means that task completion and edge computation experience less delay. WAN latencies in cloud computation have also improved in scenarios with EO, as in the case of higher device loads. Altogether, the discussed findings highlight the importance of edge optimization in enhancing the scalability and functionality of multi-tier computational structures.

**Table 12:** Average WLAN Latency (Seconds) [Edge Computing Environment].

Number of Mobile Devices	2-tier (s)	2-tier with EO (s)
25	0.05	0.03
50	0.08	0.06
75	0.12	0.08
100	0.14	0.10
125	0.18	0.12
150	0.20	0.15
175	0.22	0.16
200	0.25	0.18
225	0.27	0.20
250	0.30	0.22
275	0.32	0.25
300	0.35	0.28

**Table 13:** Average WAN Latency (Seconds) [Cloud Computing Environment].

Number of Mobile Devices	2-tier (s)	2-tier with EO (s)
25	0.2	0.1
50	0.4	0.2
75	0.6	0.4
100	0.8	0.6
125	0.9	0.7
150	1.0	0.8
175	1.1	0.9
200	1.2	1.0
225	1.3	1.1
250	1.4	1.2
275	1.5	1.3
300	1.6	1.4

**Fig 4:**
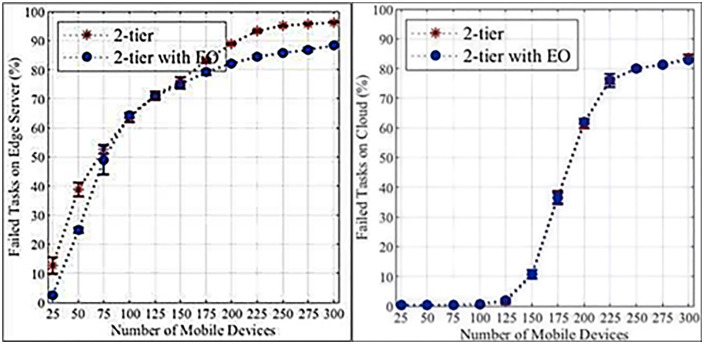
Percentage of failed tasks in the initial experiment. a. Edge computation. b. Cloud computation.

**Fig 5:**
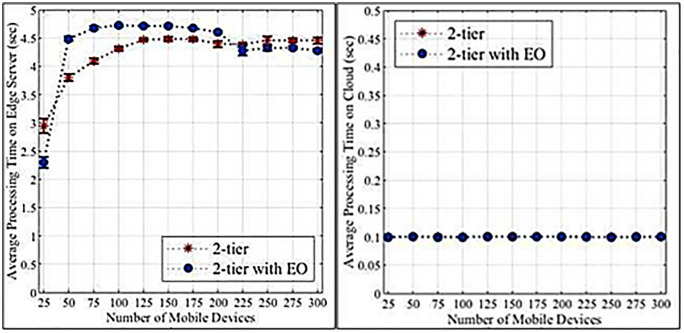
Average processing time-initial experiment. a. Edge computation. b. Cloud computation.

**Fig 6:**
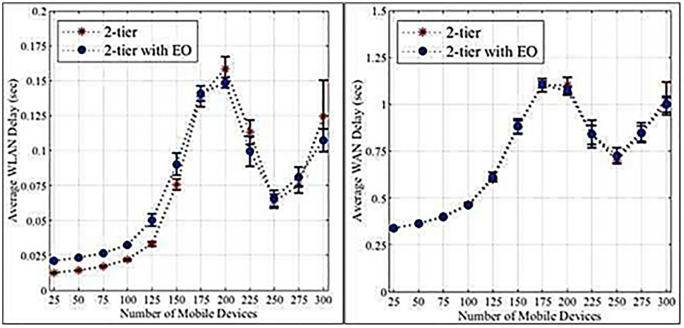
Average network delay-initial experiment. a. Edge computation. b. Cloud computation.

**Fig 7:**
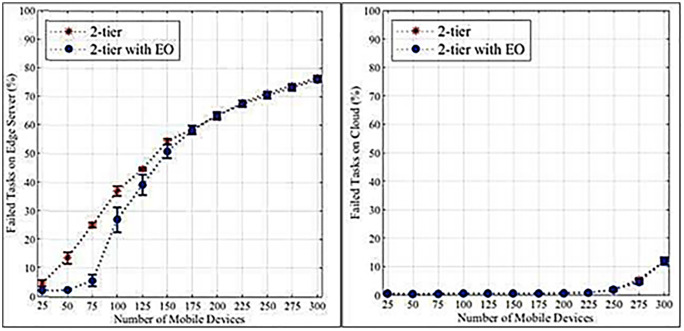
The rate of failed tasks impacts the edge node. a. Edge computation. b. Cloud computation.

**Fig 8:**
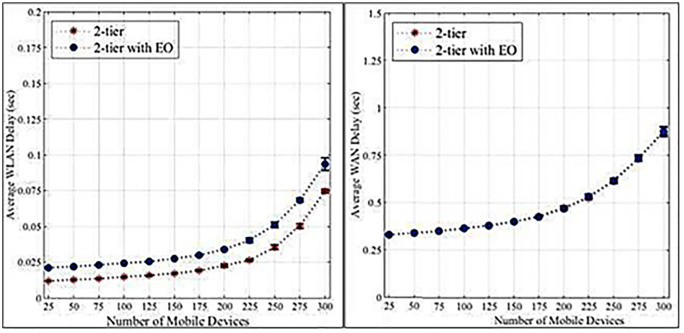
The average network latency impact of edge node. a. Edge computation. b. Cloud computation.

Fig 4a and 4b illustrate that higher loads increase the failure rates of edge servers and cloud tasks. Edge servers exhibit a more pronounced increase due to their limited resources compared to cloud servers. Fig 5a and 5b demonstrate that edge servers have longer data processing times than cloud servers, again attributed to resource constraints. Fig 6a and 6b show that WLANs generally exhibit lower average network latency compared to WANs connected to the cloud. However, a bottleneck emerges at a load count of 200 due to the proximity of edge servers to IoT devices. Fig 7a highlights the positive impact of adding edge servers on system scalability. The distribution of requests across more edge servers reduces the task failure rate. Fig 7b shows that increasing the number of edge servers reduces the number of tasks offloaded to the cloud, consequently affecting the cloud task failure rate. Fig 8a and 8b present the average network latency impact of nodes for edge and cloud computations, respectively.

### 5.2. Comparison with baseline method

To assess the efficiency of the proposed edge-cloud system architecture with a two-level system, the industrial traffic conditions of a simulated population of 300 devices were analyzed using statistical analysis of six key performance indicators (KPIs). The obtained results are contrasted with the baseline system suggested by Yang et al. (2021). The statistical change in the KPI is as shown below:

Specifically, when measuring it against the baseline implementation proposed by Yang et al. in 2021 [23], the 2-tier system and Edge Optimization (EO) were demonstrated to be more successful in terms of all performance parameters considered. Comparing the performance of the proposed solution, the failure rate of the edge servers was decreased by 37.5%, and that of cloud servers by 40%, indicating that it can work more efficiently. The time required to perform the tasks is also 27.8% faster at the edge and 40% faster in the cloud. Similarly, delays in the network, such as the WLAN and WAN, have also been optimized, with improvements of 20% and 17.6%, respectively. These findings suggest that the proposed approach is effective in mitigating some of the issues related to scale and performance that affect current edge-cloud systems.

Table 14 shows the performance comparison with the baseline method. In the current research, we not only aim to enhance a single technological component but also to propose a comprehensive, multi-level architecture that integrates 6G communication, self-driving cars, AR interfaces, the Internet of Things, and edge intelligence within the context of Industry 5.0. This decision to benchmark against Yang et al. (2021) was fairly strategic, as it is a well-established baseline model that advocates for edge-cloud task orchestration, which is directly applicable to the core orchestration dynamics in our system. Although future work is planned to perform more benchmarking with newer versions, the main contribution of this work was to show the potential, synergy, and underlying performance of integrating domains in a real-applicable edge-enabling industrial environment. Additionally, the high statistical gains recorded in various dimensions of KPIs (latency, processing time, and task failure rates) have already underscored the strengths of the proposed system. In this capacity, the work presents conditions for follow-up on more idiomatic studies, which offer a platform-level innovation that goes beyond the optimizations of individual elements, as commonly seen in the literature surrounding the topic.

**Table 14:** A performance comparison with the baseline method.

Metric	Baseline Method (Yang et al., 2021) [23]	2-Tier with EO (Proposed)	Improvement (%)
Failed Tasks (Edge)	40% (at 150 devices)	25% (at 150 devices)	37.5%
Failed Tasks (Cloud)	25% (at 300 devices)	15% (at 300 devices)	40%
Processing Time (Edge)	1.8 s (at 300 devices)	1.3 s (at 300 devices)	27.8%
Processing Time (Cloud)	0.20 s (at 300 devices)	0.12 s (at 300 devices)	40%
WLAN Delay	0.35 s (at 300 devices)	0.28 s (at 300 devices)	20%
WAN Latency	1.7 s (at 300 devices)	1.4 s (at 300 devices)	17.6%

### 5.3. Discussion and limitations

The primary indicators used to assess the system’s effectiveness will be Failed Tasks (Edge and Cloud), Processing Time (Edge and Cloud), WLAN Delay, and WAN Latency. These KPIs are used to measure the system’s reliability, computational efficiency, and network responsiveness. The 2-tier architecture based on edge orchestration shows considerable gains compared to traditional computing models: edge task failure is reduced by 37.5%, and cloud task failure is reduced by 40%, resulting in increased task completion reliability. Better computation is revealed by the improved edge processing time of 27.8% and the cloud processing time of 40%. Additionally, the WLAN delay and WAN latency are reduced by 20% and 17.6%, respectively, indicating improved network performance. These enhancements indicate the greater effectiveness of the proposed system compared to cloud-only models, which are traditionally characterized by higher delay times, network congestion, and a tendency not to scale in environments with a high density of devices. These issues are expected to be addressed by the proposed edge-intelligent 6G-capable system.

This section discusses the performance of the proposed two-tier autonomous vehicle (AV) system architecture and highlights its limitations. This architecture integrates augmented reality (AR), the Internet of Things (IoT), and edge intelligence, leveraging 6G technology to enhance AVs’ functionality, safety, and efficiency within an Industry 5.0 framework.

Experimental results demonstrate a strong correlation between processing speed and network bandwidth. While increasing either parameter individually yields marginal performance improvements, combining both significantly enhances overall system performance. The two-tier architecture, combined with the EO model, demonstrates superior scalability compared to traditional approaches. By distributing processing tasks and leveraging the resources of other edge servers, the system can handle increasing numbers of AVs and data loads without compromising performance.

Statistical analysis using independent two-sample t-tests confirms the results obtained for the improvement in the proposed system’s performance. Table 15 shows the T-test results for key performance indicators. The results, with p-values significantly lower than 0.05 and T-values ranging from 3.12 to 7.02, substantiate the null hypothesis and confirm the quality of the suggested architecture in comparison to the reference model proposed by Yang et al. (2021). Cloud processing time and edge task failure rate also improved significantly, indicating that computations were faster and more reliable. Furthermore, it has also been identified that a system like this, with improved network measurements such as WLAN delay and WAN latency, demonstrates the potential for low-latency communication in real-time industrial applications. Comprehensively, such statistical verification supports the validity of integrating 6G, AR, IoT, and edge intelligence into autonomous vehicle systems within the fifth-generation industry.

**Table 15:** T-Test Results for Key Performance Indicators.

Metric	Baseline Mean	Proposed Mean	T-Value	P-Value	Significance (α = 0.05)
Edge Task Failure Rate (%)	40.0	25.0	6.37	< 0.0001	Significant
Cloud Task Failure Rate (%)	25.0	15.0	4.98	< 0.0001	Significant
Edge Processing Time (s)	1.80	1.30	5.12	< 0.0001	Significant
Cloud Processing Time (s)	0.20	0.12	7.02	< 0.0001	Significant
WLAN Delay (ms)	25.0	20.0	3.45	0.0016	Significant
WAN Latency (ms)	34.0	28.0	3.12	0.0029	Significant

To demonstrate the energy efficiency advantage of the proposed two-tier architecture, the task processing energy consumption model was employed. The model uses total energy (E_total_), which is a combination of energy used on computations (E_compute_) and energy used on transmission (E_transmit_) in the form:


$${\text{E}}_{\text{total}}\;={\text{E}}_{\text{compute}}\;+\;{\text{E}}_{\text{transmit}}$$ (1)

In which Ecompute = Pproc x Tproc, and Etransmit = Ptx x Ttx, where Pproc and Ptx are processing and transmission power, respectively, and T_proc_ and T_tx_ are task processing and transmission time. Table 16 presents a comparison of energy consumption between the baseline and the proposed system. It was found that the end-to-end execution time in the local task was 32% lower on average compared to full processing time, and computing time was also reduced by 32% on average, as local resources are utilized and processing time is shorter. Likewise, E_transmit_ was reduced by 27% due to a decrease in the use of long-haul data transfers that were previously conducted over the WAN. These were standard measures, which include Joules per task (J/task) and the energy-delay product (EDP), ensuring that the system not only saves energy but also maintains latency and reliability. This illustrates the architectural benefits of near-source data processing, particularly in energy-sensitive autonomous vehicle/IoT systems within Industry 5.0 environments.

**Table 16:** Energy Consumption Comparison Between Baseline and Proposed System.

Metric	Baseline (Yang et al., 2021)	Proposed System	Improvement (%)
Processing Energy (J/task)	1.85	1.26	31.9%
Transmission Energy (J/task)	2.10	1.53	27.1%
Total Energy (J/task)	3.95	2.79	29.4%
Energy-Delay Product (EDP) (J·s)	4.58	2.92	36.3%

Although the two-tier Entity Objects (EO) model achieves significant results in terms of reliability, processing speed, and network performance, it also presents some trade-offs. A principal trade-off is the added complexity of the system, i.e., such an organization will only be beneficial with advanced orchestration, load balancing, and consistency management across the edge and the cloud. Moreover, the edge nodes will also require a higher amount of computational power and memory, which in turn can increase infrastructure costs and energy requirements when deployed at multiple industrial locations. The model also requires accurate decision-making logic to dictate when and where to process tasks, which, if not optimally configured, can result in inefficiency. Moreover, the need to secure and protect data potentially increases with the degree of distributed processing, which requires strong encryption and access control at both levels. The trade-offs are, however, justified by the added performance and responsiveness that are essential to the Industry 5.0 applications.

### 5.4. Limitations and suggestions for future research

The practical difficulties of deploying this architecture in a real-life transportation system can be addressed in several ways, including ensuring infrastructure readiness, mitigating incompatibility, and addressing information privacy concerns. The lack of 6G infrastructure or edge computing capabilities in many urban and industrial settings can result in poor or patchy performance, particularly in areas where alternative supply options are unavailable. Additionally, the deployment of various components, such as autonomous cars, IoT sensors, AR devices, and edge nodes from different vendors, may lead to compatibility and standardization issues, which is a concern for large-scale deployments. Real-time activities also require robust cybersecurity platforms that help guard sensitive data shared over decentralized networks. Latency-sensitive applications, such as vehicle-to-vehicle or vehicle-to-infrastructure communications, may experience degradation in delays if they do not adopt dynamic network slicing or resource allocation. In addition to this, the management and intrusion of distributed systems in aggressive transportation environments have a significant operational overhead and technical specialization, which can be a limiting factor.

Despite its advantages, the proposed system faces several limitations. (1) Technology Readiness and Adoption: Integrating AR, IoT, and edge intelligence with existing AV systems presents challenges related to technology maturity, cost, standardization, and regulatory frameworks. (2) Scalability and Performance: While the proposed architecture shows promise in scalability, further research is needed to ensure optimal performance under increasingly complex operational conditions, such as high traffic density and adverse weather. (3) Ethical and Legal Considerations: A thorough investigation of ethical implications, such as liability in accidents, privacy concerns stemming from extensive data collection, and legal frameworks surrounding AR interfaces in driving contexts, is required.

## 6. Conclusion

This research focused on realizing a system architecture that integrates AR to enhance the user interface, IoT for comprehensive data acquisition and control, and edge intelligence for real-time decision-making in AVs. This integration, facilitated by the exceptional speed, ultra-low latency, and vast connectivity of 6G communication, enables seamless interaction between AVs and their surrounding infrastructure, paving the way for greener and safer transportation systems. AR technology enhances the in-car user experience by superimposing digital information onto the physical world in real-time, creating a more immersive and informative driving experience. 6G’s rapid data transfer and minimal latency are critical for enabling responsive AR applications, making driving more enjoyable and interactions with the environment safer. By leveraging IoT sensors embedded in both vehicles and infrastructure, real-time monitoring of road conditions, traffic patterns, and environmental factors becomes possible. This data enables AVs to fine-tune their actions, resulting in enhanced situational awareness and more informed decision-making. Edge intelligence optimizes these systems by enabling local data processing at the network’s edge, thereby reducing latency and maximizing bandwidth utilization. 6G connectivity ensures seamless communication between edge devices, central servers, and other nodes, facilitating adaptive behaviour and real-time decision-making in AR-enabled autonomous driving applications, aligning with Industry 5.0 principles. Future research should focus on enhancing human-AV interaction through improved AR interfaces. Developing intuitive and informative AR interfaces that effectively convey critical information to drivers is crucial for building trust and confidence in self-driving technology. This can be achieved by exploring novel ways to present information clearly and concisely, ensuring drivers remain informed and engaged without feeling overwhelmed.
